# Maxillofacial surgery beyond the perfect storm of COVID-19

**DOI:** 10.1186/s40902-021-00293-8

**Published:** 2021-02-22

**Authors:** Tae-Geon Kwon

**Affiliations:** Department of Oral and Maxillofacial Surgery, School of Dentistry, Kyungpook National University, 2177 Dalgubeol-daero, Jung-gu, Daegu, 41940 Republic of Korea

After the first report of SARS-CoV-2 virus infection in December 2019, the outbreak of acute respiratory syndrome, coronavirus disease 2019 (COVID-19) affected national and global health care system and economy. The World Health Organization (WHO) declared the COVID-19 outbreak as a global pandemic on March 2020 [1]. The respiratory droplets of COVID-19 patients can be a direct or indirect source of person-to-person transmission. In dental office, there are a lot of face-to-face communications for treatments and the possibility of aerosol formation that contain patients’ saliva, blood, and other oral fluids. The SARS-CoV-2 can be persisted on the surface of instruments of days and in aerosols for hours [2]. Moreover, COVID-19-positive patients without signs or symptoms of COVID-19 also can transmit the disease, the exact scientific data on SARS-CoV-2 viral transmission during dental procedure [3]. However, aerosols generated from dental procedures in COVID-19 patients also can contain SARS-CoV-2 virus and potentially transmit the virus to the practitioners and other patients [4]. It is the standard recommendation to wear proper personal protective equipment (PPE) including a surgical mask, face shield, protective gown, cap, and gloves during dental procedure contacting or splashing the body fluid or blood [5, 6]. Especially for the aerosol-generating procedures, respiratory protection with N95 or FFP2 respirator is recommended because it can filter the droplet and protect exhalation [3, 5]. Therefore, WHO and most of the other countries recommended to delay non-urgent oral health care. Urgent or emergency care can be provided in patients with infection, swelling, bleeding, trauma, or severe pain that cannot be controlled with routine analgesics [1, 3]. Therefore, maxillofacial surgeries such as orthognathic surgery, cleft, or reconstructive surgeries were not a primary target of treatment during the COVID-19 outbreak period. The COVID-19 pandemic completely changed the pattern of the treatment for elective surgery cases. During the severe outbreak period of COVID-19, from March to April 2020 in Korea, University hospitals in the author’s city were fully occupied with critical care for severe COVID-19 patients. Therefore, it was difficult to carry out maxillofacial surgery because of the insufficiency in assisting nurses at the operation room. In the future, COVID-19 would be controlled by a variety of efforts and development of vaccination.

The current question is “how can we perform maxillofacial surgery in the post-pandemic era?” It is not likely to return to the pre-pandemic health care environment because we do not have enough knowledge on the safety of a routine dental treatment or elective surgery on the aerosol-mediated transmission of COVID-19. It is interesting that even with the fundamental risk of transmission of COVID-19 via dental procedures, the reported case of transmission is not frequent. According to a survey on 2195 US dentists, 355 were tested for SARS-CoV-2 and 20 dentists were confirmed or probable COVID-19 infection. In most of the COVID-positive dentists (75%, *n* = 15), a probable source of infection was not identified and 5 patients were related with community transmission [7]. From an Italian report, the transmission between the patient and dental practitioners or assistants was not found after single or multiple dental consultations after the lockdown of a city [8]. A recent case report showed that even for a confirmed SARS-CoV-2-positive patient, there was no viral SARS-CoV-2 RNA was detected in oral mucosa via PCR assay [9]. Based on these reports, more prospective studies need to be performed to clarify the real risk of transmission of COVID-19 under the current protocol for disease prevention.

In the author’s hospital, from March 2020, every patients were tested for SARS-CoV-2 before admission for surgery. Emergency patients who could not perform a COVID-19 test before surgery were regarded as potential COVID-19 patients. Therefore, the elective oral and maxillofacial surgery could be selectively performed under general anesthesia. Now we are returning to a new normal. A review of current COVID-19 prevention strategies and cases of clinic-based transmission of COVID-19 need to be more thoroughly investigated.
